# Prevalence of Positive Rapid Antigen Detection Test for Group A Streptococcus Among Patients With Respiratory Symptoms in Eastern Province, Kingdom of Saudi Arabia

**DOI:** 10.7759/cureus.48607

**Published:** 2023-11-10

**Authors:** Nadira A Al-Baghli, Ahmed Al Saif, Shorok A Al Dorazi, Ammar Y Bukhamseen, Mohammed Al Eithan, Amira R Albannai, Zainab A Buhaliqa, Montaser A Bu-Khamseen, Bothaina Alyousef, Ali A Rabaan

**Affiliations:** 1 Public Health Administration, Dammam Health Network, Dammam, SAU; 2 Preventive Medicine, Eastern Health Cluster, Dammam, SAU; 3 Medicine and Surgery, King Fahd Hospital of the University, Dammam, SAU; 4 Family Medicine, Ministry of Health Holdings, Al-Hofuf, SAU; 5 Virology, Dammam Medical Fitness Center, Dammam, SAU; 6 Family Medicine, Dammam Health Network, Dammam, SAU; 7 Family Medicine, Al Ahsa Family Medicine Academy, Al-Hofuf, SAU; 8 Molecular Microbiology, Johns Hopkins Aramco Healthcare, Dhahran, SAU; 9 Public Health and Nutrition, College of Medicine, Alfaisal University, Riyadh, SAU; 10 Public Health and Nutrition, The University of Haripur, Haripur, PAK

**Keywords:** group a streptococcal pharyngitis, saudi arabia, urti, radt, centor score

## Abstract

Background

During the COVID-19 pandemic, there was a dramatic upsurge in the prevalence of respiratory symptoms, which may have altered the usual pattern of bacterial infections and relevant decision-making.

Objectives

This study aimed to investigate the prevalence of rapid antigen detection test (RADT) positivity for group A *Streptococcus* (GAS) in patients with respiratory symptoms and signs during the COVID-19 pandemic. In addition, we evaluated the association between a positive test and the modified Centor criteria in a population of children and adults with upper respiratory tract infections (URTIs).

Methods

A prospective study was conducted in primary health care centres (PHCCs) and the paediatric emergency department (ED) of the Maternity and Children Hospital in Dammam City, Kingdom of Saudi Arabia (KSA). Trained physicians collected data from patients aged three years and older or their guardian(s) regarding URTI symptoms. The modified Centor score was calculated, and RADT was performed for all patients.

Results

Data were collected from 469 patients. The prevalence of positive RADT was 19 (4.1%), and the setting was associated with RADT positivity, as 14% of ED visitors tested positive compared with 0.6% of PHCC visitors. The RADT results had an area under the curve of 0.856 (95% confidence interval (CI)=0.774-0.939), with Centor scores of 2 and 3 having a sensitivity of 89.5%/78.9% and specificity of 70.6%/80.8%, respectively. Individuals with a score of 5 had the highest rate of positive RADT (33.3%, P<0.001); a score less than 0 excluded the possibility of GAS infection.

Conclusion

The Centor score can improve effective antibiotic prescribing; however, Centor scores ≥2 should be supplemented with an additional confirmatory test. The high specificity of RADT makes it a useful tool in preventing the prescription of unneeded antibiotics.

## Introduction

Beta-haemolytic bacteria belong to Lancefield serogroup A, and they are known as group A streptococci (GAS). GAS infection is associated with a spectrum of diseases, including pharyngitis, tonsillopharyngeal abscess, impetigo, pneumonia, otitis media, sinusitis, and meningitis, in addition to post-infection complications, such as rheumatic heart disease, poststreptococcal glomerulonephritis, and peritonsillar abscess [1-5].​ Worldwide, 18 million individuals are estimated to be affected by a serious GAS disease [1].​

GAS is the most common cause of bacterial pharyngitis in children and adults [6].​ The prevalence of GAS is 37% in children who complain of sore throat, whereas in children without pharyngitis, the carriage of GAS is 12% [6].​ In adults, GAS is the aetiological agent in up to 15% of sore throat cases [7].​

There are scarce and inconsistent data on the rate of GAS in the general population of the Kingdom of Saudi Arabia (KSA) as the country has a range of geographic terrains and climates. In Makkah, the GAS rate is 40% in children with acute tonsilitis and pharyngitis and 3% in healthy children [8].​ Another study in Makkah reported a GAS carrier rate of 1.5% in 2014 [9].​ A study from Riyadh reported a minimal change in the invasive GAS disease pattern over 28 years in admitted patients [5].​ In the Eastern region, there is a relatively high incidence of rheumatic fever, and 33% of cases are recorded between November and December, which coincides with the increased prevalence of upper respiratory tract infections (URTIs) during these months [10].

The Centor score was developed to assist clinicians in discriminating GAS pharyngitis from more common viral aetiologies and in clinical decision-making. The original study (Centor et al., 1981) [11] was modified (McIsaac et al., 1998) [12] and validated (McIsaac et al., 2004) [13] to more precisely reflect the epidemiology and factors to take into consideration for individuals who present with a sore throat. The guideline recommends a rapid antigen detection test (RADT) for patients with a modified Centor score of 2 or 3; for a score ≥4, the physician may consider antibiotic prescription.

Regarding the accuracy of RADTs, a significant variability in RADT performance has been reported in different studies. A meta-analysis was conducted to assess the accuracy of RADTs for diagnosing GAS compared to two major types of tests, enzyme immunoassays and optical immunoassays, via indirect and direct comparisons. The sensitivity of the two tests was comparable (summary sensitivity 85.4% versus 86.2%, respectively). When compared with a throat culture, the RADT was highly specific to prevent unnecessary antibiotic prescription, with sensitivity and specificity of 85.6% (95% confidence interval (CI)=83.3-87.6) and 95.4% (95% CI=94.5-96.2), respectively [14].​

This study aimed to prospectively evaluate the prevalence of positive RADTs for GAS in patients with respiratory signs and symptoms during the COVID-19 pandemic in Dammam, KSA. In addition, the association between a positive RADT and modified Centor criteria was evaluated in a population of children with URTIs.

Rationale of study

Pharyngitis is a common reason for seeking medical advice in primary health care centres (PHCCs) and emergency departments (EDs). Bacterial pharyngitis is most often caused by GAS. Early diagnosis and proper management of GAS prevents complications and reduces the number of antibiotic-resistant organisms. Furthermore, emerging diseases, such as COVID-19, can present as pharyngitis, which makes early diagnosis crucial. Early diagnosis of GAS can be achieved using RADTs; however, these tests are not available in most healthcare settings in the KSA. Furthermore, there are too little data on the prevalence of GAS in the KSA to make evidence-based recommendations for providing rapid test kits to PHCCs and EDs.

Expectation of the study

The findings of this study will improve awareness of the necessity of using RADTs in decision-making for URTIs with a suspected bacterial origin.

## Materials and methods

A sequential sample was collected prospectively from the ED of Maternity and Children Hospital (MCH), which is a secondary care hospital in Dammam, KSA, and from three PHCCs in the same city. The study population comprised all individuals presenting to PHCCs in the Dammam region and the ED at the MCH in Dammam. The inclusion criteria were individuals aged three years and older who presented to PHCCs or the ED with a complaint of upper respiratory tract symptoms, such as sore throat, congested or runny nose, headache, body ache, and cough. The exclusion criteria were patients younger than three years of age, without respiratory symptoms, and those who refused to participate in the study.

Setting

The Dammam region consists of 21 PHCCs. This study was conducted at three PHCCs, Ghornata, ALazeezia, and Almobarkea, in addition to the ED at the MCH. The study was conducted from March 13, 2022 to September 30, 2022. The study population comprised all patients with respiratory symptoms who presented to the healthcare facilities and agreed to participate in the study by providing informed consent. Informed consent was obtained from adult patients (≥18 years) or the guardians of patients younger than 18 years of age. Possible risks and discomfort were discussed with the patients and/or their guardian(s). A representative sample was chosen and studied according to the criteria described below.

Data collection

The participants were asked by a well-trained physician about their wellness and ability to contribute to the study and provide informed consent. A validated questionnaire was completed during the interview with the participants. A standard throat swab was performed using polyester-tipped (Dacron) swabs. The patient was asked to tilt their head back and open their mouth wide. A healthcare provider rubbed a sterile swab along the back of the throat, near the tonsils, several times to improve the likelihood of detecting bacteria. Throat swabs were processed using Alere^TM^ TestPack Plus Strep A with On-Board Controls (Abbott, USA), which is a rapid immunoassay for the qualitative detection of GAS antigen from throat swab specimens.

Data processing and analysis

All variables were checked for accuracy and completeness and coded. Modified Centor scores were computed based on the criteria presented in Table 1 [13].

**Table 1:** Modified Centor scores

Criteria	Score
Age (years)	
3–14	+1
15–44	0
≥45	-1
Swelling or exudates on tonsils	Yes (+1)
Tender and/or swollen anterior cervical lymph nodes	Yes (+1)
Temperature >38 degrees Celsius (100.4 °F)	Yes (+1)
Cough present	No (+1)

Data were entered into a personal computer, and IBM SPSS Statistics for Windows, version 26 (released 2019; IBM Corp., Armonk, New York, United States) was used for data cleaning and analysis. Chi-squared or Fisher’s exact tests were used for bivariate analysis. The independent samples Kruskal-Wallis test was used to compare the medians of multiple groups. Binary logistic regression analysis was used for multivariate analysis. A receiver operator characteristic curve was plotted to identify the best Centor score cut-off in terms of sensitivity and specificity for a positive RADT. A p-value <0.05 was considered statistically significant.

Ethical consideration

The questionnaire and protocol were approved by the Dammam Medical Complex (DMC), KSA, Institutional Review Board (IRB) (approval no.: H-05-D-107) and by the relevant authorities before conducting the study. The aim of the study was explained to the selected population. Prior to their participation in the study, consent was obtained from the patients or, in the case of children, from their parents or guardians. If children were able to communicate, their approval for participation was obtained. All names and data were handled with respect for confidentiality.

## Results

From March 13, 2022 to September30, 2022, data were collected from 469 patients who presented to the PHCCs or ED with respiratory symptoms. Three-quarters (n=347, 74%) of the participants were recruited from PHCCs, more than one-half of the participants were female (n=247, 53%), and most participants were Saudis (n=446, 96%). Furthermore, most participants were not current smokers (91%) and had no comorbidities (81%) (Table 2).

**Table 2:** Characteristics of the study group based on the rapid antigen detection test (RADT) result (n=469) *PHCCs: primary health care centres, **ED: emergency department

Characteristics	Total n=469	Rapid antigen test result		P value*
		Positive n=19	Negative n=449	
Setting – n (%)				
*PHCCs	347 (74)	2 (0.6)	345 (99.4)	<0.001
**ED	121 (26)	17 (14)	104 (86)	
Gender – n (%)				
Female	247 (53)	12 (4.9)	235 (95.1)	0.483
Male	220 (47)	7 (3.2)	213 (96.8)	
Nationality – n (%)				
Saudi	446 (96)	18 (4)	428 (96)	1.000
Non-Saudi	21 (4)	1 (4.8)	20 (95.2)	
Comorbidities – n (%)				
Yes	89 (19)	4 (4.5)	85 (95.5)	0.768
No	379 (81)	15 (4)	364 (96)	
Current smoker - n(%)				
Yes	44 (9)	0 (0)	44 (100)	0.240
No	422 (91)	19 (4.5)	403 (95.5)	

Overall, the prevalence of a positive RADT was 4.1% (19/469). Among the tested background variables, only the setting was significantly associated with RADT positivity, as 14% of ED visitors tested positive compared with 0.6% of PHCC visitors (Table 2). Multivariate analysis was performed to evaluate the predictors of positive RADT and included a history of receiving antibiotic treatment in the last 28 days and Centor criteria (i.e., sore throat, tonsillar exudate, lymph node swelling, age group, and absence of cough). Both a history of sore throat and the presence of tonsillar exudate increased the odds of having a positive RADT by approximately 10-fold (sore throat: OR=9.89, 95% CI=2.7-36.23; exudate: OR=9.98, 95% CI=2.13-46.71). Compared with the younger age group (three to 14 years), adolescents and adults (15-44 years) had 89% reduced odds of having a positive RADT (OR=0.11, 95% CI=0.01-0.95). None of the older adults (≥44 years) tested positive. The other Centor criteria and a history of receiving antibiotics were not significantly associated with a positive RADT (Table 3).

**Table 3:** Multivariate analysis for predictors of a positive rapid antigen detection test (RADT)

Variable	Adjusted OR (95% CI)	P value
History of receiving antibiotic treatment within the last 28 days	0.8 (1.24–0.24)	1.000
Sore throat	9.89 (2.7–36.23)	0.001
Fever	2.96 (0.48–18.46)	0.245
Tonsillar exudate	9.98 (2.13–46.71)	0.003
Lymph node swelling	0.98 (0.22–4.41)	0.975
Absence of cough	1.36 (0.44–4.19)	0.597
Age group (years)		
3–14	Reference	
15–44	0.11 (0.01–0.95)	0.045
≥44	0	0.996

**Figure 1:**
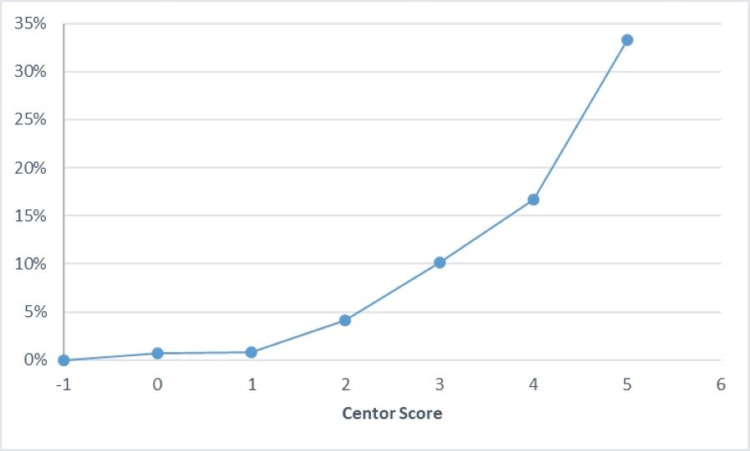
Percentage of positive RADTs according to the Centor score

The percentage of positive RADTs increased as the Centor score increased (Figure 1).

**Figure 2:**
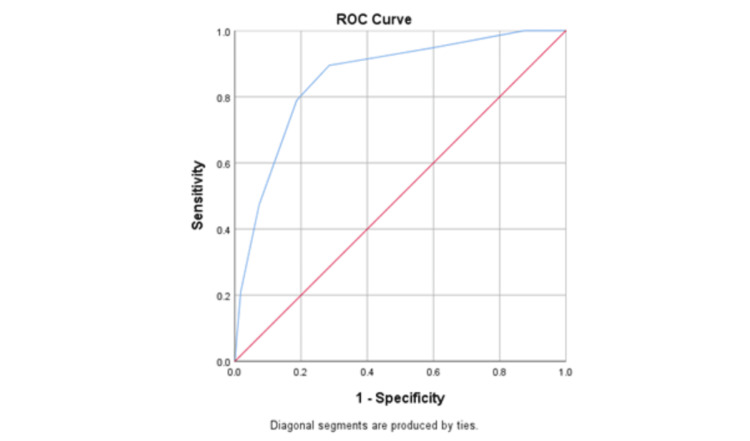
Performance of the Centor criteria in predicting positive rapid antigen detection tests (RADTs) ROC: receiver operating characteristic

Centor scores performed relatively well in classifying RADT results, with an area under the curve of 0.856 (95% CI=0.774-0.939) (Figure 2).

The highest Youden index (J), which indicates the maximum vertical distance from the curve to the chance line, corresponded to Centor scores of 2 and 3, with a sensitivity of 89.5%/78.9% and specificity of 70.6%/80.8%, respectively (Table 4).

**Table 4:** Sensitivity, specificity, and Youden index values for each Centor cut-off score predicting rapid antigen detection test (RADT) positivity

Positive if greater than or equal to:	Sensitivity	Specificity	J
-1.0	100	0	0.000
0.0	100	12.5	0.125
1.0	94.7	40.1	0.348
2.0	89.5	70.6	0.601
3.0	78.9	80.8	0.597
4.0	47.4	92.7	0.401
5.0	21.1	98.2	0.193

## Discussion

The prevalence of positive RADTs among patients presenting to the ED and PHCCs with symptoms and signs of URTI was 4.1%. This relatively low prevalence may have been related to the increased incidence of COVID-19 during the study period, which may have diluted the prevalence of non-COVID-19 URTIs and may not reflect the usual pattern of bacterial infections. It is estimated that the prevalence of positive RADTs is significantly higher among those visiting the ED in a paediatric hospital. This can be explained by the more severe symptoms of patients visiting the ED compared with PHCCs, which is reflected by the higher Centor score in the paediatric group. Considering this observation, physicians should be provided with tools to guide them in the diagnosis of bacterial infections to ensure that patients receive appropriate management with rational prescription of antibiotics to avoid complications due to streptococcal infection.

This study is unique as it identified the cut-off point for a local population in the KSA during the COVID-19 pandemic compared to the Centor criteria, with a trade-off between sensitivity and specificity. Centor scores of 2 and 3, with a sensitivity of 89.5%/78.9% and specificity of 70.6%/80.8%, were the optimal cut-off points for identifying a possible infection. At the lowest possible Centor score (-1), all true positive cases are identified as positive (100% sensitivity), while none of the true negative cases are identified as negative (0% specificity). As the cut-off increases, some positive cases with lower scores are missed and falsely identified as negative (lower sensitivity), while more true negative cases are identified correctly (higher specificity).

Our findings support the use of clinical scoring systems to exclude patients with Centor scores less than 2 from GAS testing. The original and validated research by McIsaac (1998, 2004) [11-13] advised performing a RADT on individuals with a modified Centor score of 2 or 3 and suggested empiric antibiotic treatment for individuals with a modified Centor score ≥4. However, according to the 2012 guidelines from the Infectious Disease Society of America, treatments should only be initiated when an antigen or culture test is positive or in the presence of specific risk indications [15].​

The modified Centor criteria provide diagnostic criteria and pre-test probability for streptococcal infection. It has been reported that cases with modified Centor scores ≥4 have a risk of 51-53% for having a GAS infection [16].​ By contrast, a score ≥4 was less associated with GAS in the present study; however, RADT positivity was higher in patients who scored 4 (16.7%) and 5 (33.3%). The lower percentage obtained in our study might be attributed to the increased prevalence of respiratory signs and symptoms during the study period due to the COVID-19 pandemic. However, these data may still reflect a significant rate of inappropriate antibiotic use related to empirical therapy in patients with Centor scores ≥4.

A meta-analysis [17] comparing the performance of the Centor and McIsaac scores revealed that both scores reasonably distinguish between patients with and without GAS infection, indicating that additional point-of-care tests are required to definitively diagnose GAS. Nonetheless, a score of ≤0 may be adequate to rule out infections, which is supported by the results of the present study, where no participants with a score of -1 had a positive test (P<0.001).

Because the RADT is still unavailable at most governmental healthcare facilities, the modified Centor criteria are a suitable solution to indicate supportive treatment without antibiotics. However, for high Centor scores, confirmation by either RADT or culture is necessary. In addition to being less expensive than culture, RADT can provide a result in less than 15 minutes, and confirming high Centor scores with RADT reduces the likelihood of disease spread, the duration of the symptomatic period, the likelihood of developing invasive complications, the amount of time missed from work or school, inappropriate use of antibiotics, patient and guardian dissatisfaction, and the need for follow-up visits [18].​

Limitations

The main limitation of this study was the inability to mask the RADT results from the physicians, which may have affected their decision to prescribe antibiotics. In addition, it was not possible to implement a systematic random selection of patients given the context of a busy clinic.

## Conclusions

Centor scores performed well in classifying RADT results, with optimal cut-off points at scores of 2 and 3, offering a balance between sensitivity and specificity. The prevalence of RADTs among patients with respiratory symptoms is relatively low, but it is more common among patients attending the emergency department. For patients with Centor scores ≥2, it is advisable to perform an additional confirmatory test to diagnose GAS pharyngitis and thereby reduce unnecessary antibiotic prescriptions.
